# Temporal Shifts in Microbial Communities in Nonpregnant African-American Women with and without Bacterial Vaginosis

**DOI:** 10.1155/2008/181253

**Published:** 2009-01-27

**Authors:** John Wertz, Natasha Isaacs-Cosgrove, Claudia Holzman, Terence L. Marsh

**Affiliations:** ^1^Department of Biology, Calvin College, 3201 Burton Street, S.E. Grand Rapids, MI 49546, USA; ^2^Department of Microbiology and Molecular Genetics, Michigan State University, East Lansing, MI 48824, USA; ^3^Department of Epidemiology, Michigan State University, East Lansing, MI 48824, USA

## Abstract

Bacterial vaginosis (BV) has been described as an increase in the number of anaerobic and facultatively anaerobic bacteria relative to lactobacilli in the vaginal tract. Several undesirable consequences of this community shift can include irritation, white discharge, an elevated pH, and increased susceptibility to sexually transmitted infections. While the etiology of the condition remains ill defined, BV has been associated with adverse reproductive and pregnancy outcomes. In order to describe the structure of vaginal communities over time we determined the phylogenetic composition of vaginal communities from seven women sampled at multiple points using 16S rRNA gene sequencing. We found that women with no evidence of BV had communities dominated by lactobacilli that appeared stable over our sampling periods while those with BV had greater diversity and decreased stability overtime. In addition, only *Lactobacillus iners* was found in BV positive communities.

## 1. INTRODUCTION

The relationship between biodiversity and ecosystem stability has been critically discussed and investigated over the past decade [1–10]. Most reports have provided evidence suggesting that greater biodiversity leads to greater system stability in the face of stress. A number of theoretical constructs have been created to account for the relationship between biodiversity and stability [3, 11, 12] in which high biodiversity within an ecosystem is frequently equated with a level of functional redundancy. Thus in periods of stress, the loss of a species is not catastrophic given a level of redundancy.

Microbial communities provide a remarkable system for investigating these relationships. Many apparently stable microbial communities are constructed of hundreds or thousands of species. Notable examples are the human intestinal microflora with an estimated 500–600 species [13, 14] and soil with an estimated 2000–3000 species/gram [15]. Perturbations resulting in significant community shifts have been detected in both of these communities (e.g., [16]), but their stability has not been carefully measured nor has the level of biodiversity been robustly correlated with stability. Interestingly, Fernández et al. [17] described a bioreactor with functional stability but apparent dynamicism in the phylogenetic composition of the community throughout the experiment. This is consistent with a level of functional redundancy among the species present that maintained the overall process in spite of phylogenetic shifts within the community.

From the perspective of the biodiversity-stability debate, the vaginal tract is an interesting ecosystem. In a large percentage of females the vaginal microbial community is relatively simple and dominated by one or several species of *Lactobacillus* [18–23]. However, when this simple community is replaced by bacterial vaginosis (BV), the shift is from the near monoculture of lactobacillus to a community with orders of magnitude greater phylogenetic diversity, especially in regards to Gram-positive anaerobes [19, 24–27]. Only a few investigators have addressed the stability of the community over time for either BV negative or BV positive females (e.g., [28, 29]).

In the work described herein, we present phylogenetic assessments of the vaginal microbial community from nonpregnant women. Multiple samples were taken from each woman on a monthly schedule, and the phylogenetic composition of the communities was determined by comparative sequence analysis of 16S rRNA gene libraries. Our goal was to compare the microbial community structure in BV positive and BV negative women over time and examine whether diversity correlated with greater stability.

## 2. METHODS

### 2.1. Study sample

Vaginal samples used in this study were collected as part of a small, randomized clinical trial (RCT) of vaginal douching cessation. The primary goals of the RCT were to assess the acceptability of douching cessation and an at home data collection protocol over a four-month period. Secondary goals included describing BV presence/absence throughout the study period, and identifying factors associated with BV (e.g., phase of menstrual cycle, lifestyle). The study was conducted on a college campus and eligibility criteria included douching currently at least once per month and not being pregnant. Women were enrolled over a six-week period and total sample size was limited to the first 45 eligible women. At enrollment, women met with study personnel at the campus clinic to review and sign consent forms and to complete a baseline questionnaire. Participants were then randomized either to continue usual douching patterns or to refrain from all vaginal douching. At enrollment, women self-collected two swabs for baseline data on vaginal microflora. Thereafter, study participants were asked to complete a daily diary and self-collect three vaginal swabs a week (one on the weekend and two spread across the weekdays) for four months. Diaries included information about days of menses, sexual activity, contraceptive use, vaginal douching, vaginal symptoms, bathing, showering, illness, medications, and stress level. Diary sheets and slides were returned by mail weekly. Participants also returned to the campus clinic every two weeks at which time a swab for BV assessment was collected and vaginal pH was measured. Once per month an additional swab was collected, placed in sterile saline and frozen at −80°C.

For the current study, frozen vaginal fluid samples from seven women enrolled in the RCT were selected for further study. All seven women were African-American, and they had been assigned to the intervention arm (i.e., asked to refrain from vaginal douching) and reported that they did not use any form of hormone-based contraceptive. For comparison purposes, five of the seven women were selected because, throughout the four months of the RCT, all of their vaginal smears were negative for BV; the other two women were selected because they frequently showed evidence of BV during the same study period. Clone libraries of 16S rRNA genes were constructed from 2-3 monthly vaginal samples of each BV negative woman and 4 monthly samples from BV positive women.

### 2.2. Nugent scoring for BV

A single microbiologist with training in the Nugent method for scoring BV [30] evaluated all vaginal smears while blinded to the randomization assignment and data from the diaries. In a previous study with the same microbiologist and a second microbiologist, the kappa for BV positive (Nugent score ≥7) versus BV negative smears was .81 [31]. Nugent scores range from 0 to 10, the higher scores are indicative of more Gram-negative aerobes and Gram-positive anaerobes and fewer lactobacilli. A Nugent score of 0–3 is considered BV negative, 4–6 is intermediate, and 7–10 is BV positive.

### 2.3. Extraction of DNA and PCR amplification

Microbial DNA was extracted using MoBio Soil DNA extraction kits as follows. Frozen vaginal swabs were soaked in 70% ethanol overnight. The tip was removed from the tube and residual ethanol was squeezed out on the side of the tube. The swab tip was then cut off and placed into the MoBio extraction tube and stored at −20°C until extraction. The ethanol wash was centrifuged for 30 minutes at 10,000× g in a microfuge and the resulting pellet was resuspended in 200 *μ*L of water and transferred to the MoBio extraction tube with the swab tip. The combined pellet and swab tip were lysed by bead beating for 1.5 minutes and then extracted according to the manufacturer's instructions. This protocol ensured that both free DNA derived from lysed cells and DNA from intact cells were collected from the samples. Pilot PCR reactions of 25 *μ*L were performed using bacterial domain specific primers 27F (5′-AGA GTT TGA TCM TGG CTC AG-3′) and 1389R (5′-AGC GGC GGT GTG TAC AAG-3′) [32]. The PCR reaction volume was 25 *μ*L with 30 ng template DNA. Reactions contained 1X buffer (Invitrogen), 1.5 mM MgCl_2_, 0.25 mM of dNTPs, and 0.2 *μ*M of each primer and 0.6 units of Taq polymerase (Invitrogen). Cycling was initiated with an initial denaturation of 3 minutes at 95°C followed by 25 cycles of 45 seconds at 95°C, 45 seconds at 56°C and 1 minute at 72°C, followed by a 5-minute extension at 72°C. PCR products were analyzed on agarose gels stained with ethidium bromide. Reactions with the appropriate size PCR product were cloned using Invitrogen's TOPO cloning kit. Putative clones with inserts were picked, screened and sequenced at the technology center at MSU. Sequences were deposited at GenBank (EF364727 to EF365525 (low Nugent scores) and EF365526 to EF366669 (high Nugent scores)).

### 2.4. Phylogenetic and statistical analyses

Each 16S rRNA gene clone was assigned a preliminary phylogenetic affiliation by sequence comparison to the Ribosomal Database Project II using the sequence match tool [33]. Sequences were checked for chimerae using the Chimera Check program [34], and sequences shorter than 550 nucleotides were removed. Sequences that were not clearly assigned at the genus level were compared to the Genbank nucleotide database using BLAST [35]. Sequences were aligned based on secondary structure to the 16S rRNA gene sequence database ssuJan03 in the ARB software package (http://www.arb-home.de/) using the Fast Aligner tool [36]. Unaligned or ambiguously aligned nucleotides were corrected manually. For all subsequent analyses, 503 unambiguously aligned nucleotides corresponding to positions 119 to 638 in *Escherichia coli* were used.

For phylogenetic analyses, when closely related sequences were not identified in the ARB database, relatives were found by a BLAST search of the Genbank database and incorporated into ARB. Phylogenetic trees were constructed using the neighbor-joining method with a Felsenstein correction. A minimum evolutionary distance method in PAUP* was used for bootstrap analysis of the same data.

Differences in the libraries were tested by pairwise comparison of PHYLIP-formatted distance matrices for each library using webLIBSHUFF version 0.96 [37], which combines preLIBSHUFF [38] and LIBSHUFF version 1.22 [39]. For further community analyses, the sequences were grouped into operational taxonomic units (OTUs) using DOTUR [39]. A distance of 3% was used to define an OTU, and is hereafter denoted as OTU_0.03_. A 3% dissimilarity in 16S rRNA gene sequences is typically, though controversially, thought to represent a species-level delineation [40]. For each participant, the two-, three-, or four-clone libraries were combined and the Chao1 richness and Simpson diversity (D) estimators were calculated as implemented in the DOTUR program. The Simpson index of diversity was calculated as 1D. The Chao1 estimator, at an OTU_0.03_ cutoff, can be thought to represent the estimated number of species in an environment. The Simpson index of diversity is an estimate that takes into account the richness as well as the evenness (number of each species). To obtain a quantitative measure of the OTU_0.03_ similarity between libraries sampled from the same participant, the Yue and Clayton nonparametric maximum likelihood method was calculated using the SONS software [41].

## 3. RESULTS

In the seven women selected for this study, vaginal pH ranged from 4.0 to 5.8 and, as expected, pH was highest when BV was present (Table 1). For most women, samples were obtained at different menstrual weeks. Among BV positive women, there were no reports of antibiotic or antifungal use, and intercourse was infrequent in the week before vaginal sampling.

We evaluated the structure of the microbial communities from the seven women described in Table 1 with 16S rRNA gene libraries. A total of 20 libraries were made from both low- (5 women and 12 libraries) and high-(2 women and 8 libraries) Nugent scoring women. A total of 1,943 sequences were analyzed with library sizes raging from 50 to 170 clones (Table 1). *Lactobacillus* was the numerically dominant genus in 17 of the libraries. Three of the libraries from high-Nugent scoring women were dominated by *Leptotrichia/Sneathia, Prevotella,* and *Megasphaera,* respectively. In total, 28 genera were detected within the Firmicutes, Bacteriodetes, Actinobacteria, Proteobacteria, and Fusobacteria phyla. In the BV positive women, 20 different genera were detected while only 14 (of which 9 were singletons) were identified in the BV negative women.

**Figure 1:**
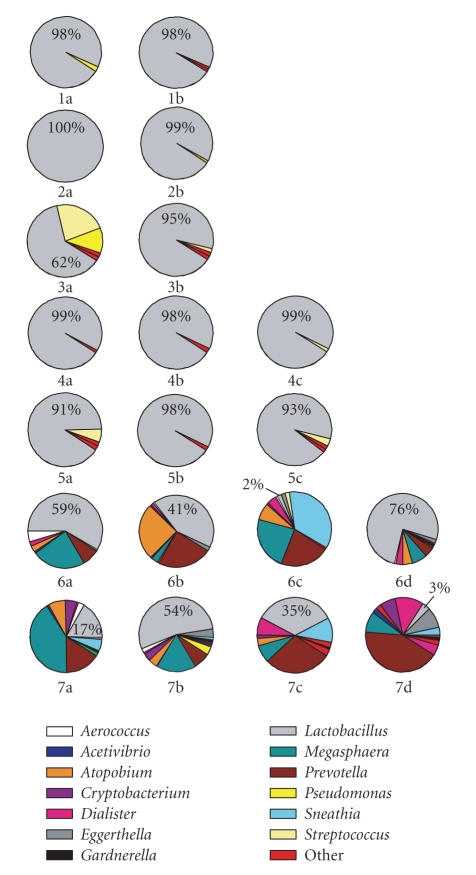
Pie chart representations of the vaginal microbial community structure between participants (top to bottom) and within each participant, over time (left to right) as inferred by 16S rRNA gene libraries. The percentage of each library consisting of clones related to members of the *Lactobacillus* genus is given.

Figure 1 presents the relative abundance of the detected genera in all of the libraries. The top 12-community composition profiles represent the communities from the five individual women with low-Nugent scores. These communities were dominated by lactobacilli which usually constituted 91% of the community or greater. The exception to this was library 3a where *Lactobacillus* constituted only 62% of the clones.

The eight libraries derived from two women with high-Nugent scores are presented in the bottom two rows of community composition profiles in Figure 1. These revealed considerably more phylogenetic diversity than that found in low-Nugent scoring communities, consistent with the morphological basis of the Nugent scoring system and previously recorded observations [30]. Ten genera were identified in these libraries that were not detected in libraries from low-Nugent scoring women. Most of these genera displayed considerable volatility over time. For example, in woman #6, the genus *Megasphaera* constituted 22%, 3%, 27%, and 10% of libraries A, B, C, and D, respectively. This irregular flux in clone numbers was also seen in *Prevotella* in woman number 7. Moreover, the lactobacilli were also volatile in clone numbers over time and were, in general, greatly reduced in numbers in women with high-Nugent scores. This is in contrast to libraries from low-Nugent scoring samples where lactobacilli were routinely high and constant in clone numbers over time.

To quantitate these diversity differences we applied a suite of ecological and statistical measurements to these libraries (Table 2). The Simpson's diversity index revealed at least a twofold difference between low and high-Nugent scoring communities while the Chao species richness similarly revealed substantial differences between these two groups. The Yue and Clayton analysis [41] measures library similarities. In this table, we calculate intra-woman library similarities and then compare these across the range of Nugent scores. All libraries with low- Nugent scores had high similarity (>79%) whereas the high-Nugent scoring libraries had low similarities (<44%). On visual inspection of these libraries, it was clear that there was structural instability in the community over time. Nugent scores did not reveal subtleties of phylogenetic composition as demonstrated by comparing community profiles 3a and 6d.

Analysis of the microbial communities among and between participants with high- and low-Nugent scores showed that approximately 95.0% (758 out of 799) of the clones from the low-Nugent scoring women were lactobacilli (Table 3). Of the remaining 5%, most were identified as streptococci (19 clones) or pseudomonads (9 clones). The genus *Lactobacillus* also contained the most number of clones of any other genus identified in the participants with high-Nugent scores, though the lactobacilli only accounted for 38.3% (438 out of 1144) of the total. A majority of the remaining clones grouped with the genera *Prevotella* (17.3%), *Megasphaera* (15.7%), *Atopobium* (7.5%), *Sneathia* (7.3%), *Dialister* (3.6%), and *Cryptobacterium* (2.4%). Of these, only *Sneathia* was not consistently present in all eight libraries (Figure 1). No clones belonging to any of these genera were obtained from participants with low-Nugent scores.

Among the two participants with high-Nugent scores, the distribution of *Prevotella*, *Atopobium,* and *Cryptobacterium* species was distinct (Figure 3). A majority of *Prevotella* clones from participant 6 grouped with *Prevotella bivia*, whereas those from participant 7 grouped most closely with *Prevotella buccalis*, *P. corporis* and *P. disiens*. Similarly, participant 6 had approximately three times the number of clones that grouped with *Atopobium vaginae* than did participant 7, whereas participant 7 had approximately three times the number of clones grouped with *Cryptobacterium curtum* than participant 6 (Figure 3). Both participant 6 and 7 had a similar overall distribution of species within the *Megasphaera* and *Dialister* genera.

**Figure 2:**
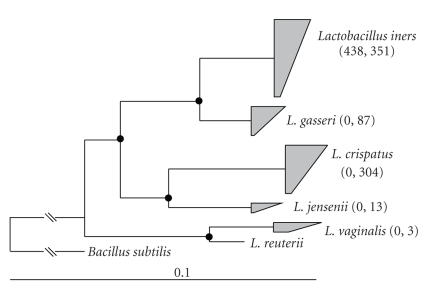
Neighbor joining based phylogeny of the 1,196 *Lactobacillus* 16S rRNA gene clones obtained in this study. Clones that were closely related to known *Lactobacillus* species were condensed into trapezia. Numbers in parentheses represent, for each group, the number of clones obtained from participants with bacterial vaginosis (left) and without bacterial vaginosis (right). The phylogeny is based on 503 unambiguously aligned nucleotides. Branch points with >75% conservation are represented with a closed circle; branch points with 50–74% conservation are shown with an open circle. Genbank accession numbers for reference species are shown in brackets. A 16S rRNA gene from *Bacillus subtilis* was used as the outgroup. Scale bar represents 0.1 change per nucleotide.

**Figure 3:**
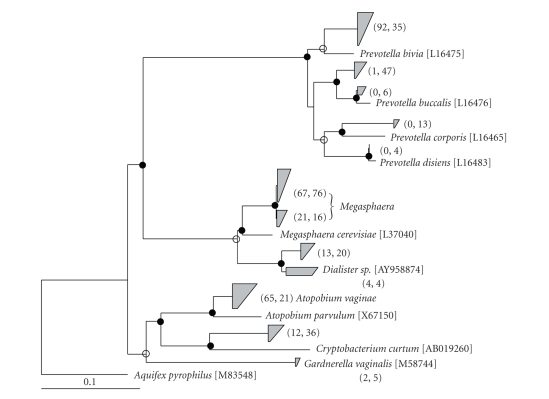
Neighbor joining-based phylogeny of 16S rRNA gene clones related to bacterial genera consistently present in participants with bacterial vaginosis. Closely related clones were condensed into trapezia with numbers in parentheses representing, for each group, the number of clones obtained from participant 6 (left) and participant 7 (right). The phylogeny is based on 503 unambiguously aligned nucleotides. Branch points with >75% conservation are represented by a closed circle. Genbank accession numbers for reference species are shown in brackets. A 16S rRNA gene from *Aquifex pyrophilus* was used as the outgroup. Scale bar represents 0.1 change per nucleotide.

As mentioned above, clones belonging to the genus *Lactobacillus* were the most abundant, irrespective of Nugent score. However, participants with low-Nugent scores had a diversity of *Lactobacillus* species that included *L. iners*, *L. gasseri*, *L. crispatus*, *L. jensenii*, and *L. vaginalis* (Figure 2) whereas libraries from participants with high-Nugent scores contained only *L. iners* (Figure 2).

## 4. DISCUSSION

Regarding the vaginal tract community structure of women with low-Nugent scores, our results were similar to previously reported studies [18–20, 23, 42]. All communities were dominated by Lactobacillus spp. Five different species were detected in the 758 *Lactobacillus* sequences including *L. iners, L. gasseri, L. crispatus, L. jensenii*, and *L. vaginalis*. Interestingly, we failed to detect any Lactobacillus sp. other than *L. iners* in BV positive women. Similar asymmetric distribution of lactobacillus species have been reported where *L. gasseri* and *L. iners* were “negatively correlated to each other” [43] or positively correlated with BV-associated bacteria [44]. Our results suggest that *L. iners* may be better adapted to the polymicrobial state of BV, including elevated pH.

Bacterial vaginosis has been described as a polymicrobial syndrome [19, 21, 25, 45, 46] with higher microbial diversity than what is perceived as the healthy ground state dominated by lactobacilli. Clinically it is characterized by a white discharge, an increase in pH and amine concentration, the appearance of clue cells, and a microbial community shift detected by Gram stain of smears from vaginal fluid [19, 21, 24–26, 45, 46]. Similar to previous work (e.g., [19, 47]) we detected greater species diversity in the BV positive subjects. In our 7 samples from the two BV positive women, we detected five clades within the *Prevotella* genus, the most abundant of the nonlactobacillus genera present in our libraries. Two of the *Prevotella* clades detected were present in both BV positive women while three were present in only one. This may reflect host differences that select for unique species or the consequences of sampling at nonsaturating levels. *Magasphaera* (2 clades, 180 clones), *Dialister* (2 clades, 41 clones), *Cryptobacterium* (1 clade, 27 clones) *Atopobium* (1 clade, 86 clones), *Eggerthella* (1 clade, 21 clones), and *Gardnerella* (1 clade, 7 clones) were also detected in BV positive women, although clone numbers were different. The surprising aspect of these studies was the volatility in clone demographics over time exhibited by the BV positive women. This suggests that in the case of women with clinically identified BV, the increase in diversity is accompanied by a decrease in community stability. It is possible that in spite of the phylogenetic volatility, the community function remains constant, as in the case of the previously cited bioreactors [17]. Other explanations are possible (see below). Nonetheless, in our BV positive women the phylogenetic composition changed dramatically over time in contrast to women with low-Nugent scores.

It is intriguing to consider the vaginal community in the light of ongoing discussions of biodiversity and stability of ecosystems, in part because of the demographic instability that we detect when the community is at its greatest diversity, in the BV positive women. While it seems (somewhat) intuitive to equate high biodiversity with a more resilient ecosystem; previous workers have concluded that there was “no such arbitrarily general rule” [48, 49]. Indeed, May points out that randomly constructed ecosystems “are more likely to lose species after disturbances than are simple ones” [49]. Moreover, in a separate paper May reported that simple nonlinear difference equations that describe growth can produce stable cycles as well as apparent chaotic regimes [50]. Hence, the community instability in the BV positive state that we observed could be more a reflection of a randomly assembled community and/or the composite of populations with nonoverlapping growth curves.

BV can be a recalcitrant condition even in the face of clinical treatment [25, 26, 46]. While the molecular approaches of microbial ecology have provided considerable insight into the phylotypes present [18–20, 23, 28, 42, 44], we remain somewhat distant from a complete ecological description of the vaginal community that includes the host genotypic variability, environmental influences, a complete description of the community including eukaryotes, bacteria and viruses [51] and critical interactions between species, not to mention prevailing nutrient sources and food webs [52]. It is encouraging that some investigators have identified strong correlations between certain bacterial phylotypes and BV (e.g., [19]). In addition, the hormonal milieu appears to influence vaginal microflora, as evidenced by a lower prevalence of BV in women exposed to exogenous hormones [31, 53] and a higher prevalence of BV in the first week of the menstrual cycle [31, 54]. In this study of seven selected participants, we specifically chose women who were unexposed to exogenous hormones and had consistent BV scores (i.e., primarily negative/intermediate or primarily positive) irrespective of the timing in the menstrual cycle. Moreover, we have come to view the syndrome as an ecosystem gone awry and currently efforts are being directed at identifying the conditions or events that cause community shifts [25, 26, 46]. While this ecosystem approach is more complex, it may prove more productive than pathogen hunting.

Our report is a preliminary study of relatively few women sampled over time where community structure was determined using culture independent techniques. We recognize the potential biases that can arise from PCR amplification and library construction [55] including primer bias. The latter is of particular concern because some phylogenetic groups can be missed entirely by poorly matched primer sets. For example Frank et al. [56] and Verhelst et al. [42] recently demonstrated that detection of *Gardnerella*, a genus frequently associated with BV (e.g., [42]), can be strongly influenced by primer selection. While our primer set did pick up *Gardnerella* sequences, the abundance may have been influenced by primer bias. However, in spite of these limitations we have identified substantial diversity within the *Prevotella* clones, an asymmetric distribution of the lactobacilli species and large demographic shifts over time in BV positive women.

## Figures and Tables

**Table 1:** Relevant clinical and 16S rRNA gene clone library information for the seven participants in this study.

Participant	Library	BV Score	pH	Menstrual cycle (week)	Frequency of intercourse (week prior)	Antibiotic/ antifungal (previous month)	No. of clones in library	Phylogenetic affiliation of dominant phylotype	Dominant phylotype (% of library)
1	1a	0	4.4	2	0	No	85	*Lactobacillus crispatus*	98
	1b	1	4.4	3	0	No	86	*L. crispatus*	98

2	2a	0	4.0	4	0	Antibiotic	50	*L. crispatus*	100
	2b	0	4.0	4	0	No	91	*L. crispatus*	99

3	3a	2	4.7	3	0	Antibiotic	53	*Lactobacillus gasseri*	62
	3b	4	4.7	3	0	No	58	*L. gasseri*	95

4	4a	1	4.4	4	4	Antifungal	79	*Lactobacillus iners*	99
	4b	2	4.7	4	0	Antifungal	50	*L. iners*	98
	4c	0	4.4	6^*#*^	1	Antifungal	74	*L. iners*	99

5	5a	0	4.0	4	3	No	53	*L. iners*	91
	5b	4	4.7	2	4	No	60	*L. iners*	98
	5c	0	∗	5^*#*^	0	No	60	*L. crispatus*	93

6	6a	8	5.0	2	0	No	147	*L. iners*	59
	6b	8	5.8	4	1	No	170	*L. iners*	41
	6c	8	5.8	3	0	No	162	*Leptotrichia amnionii*	36
	6d	4	4.0	5^*#*^	0	No	159	*L. iners*	76

7	7a	8	5.0	3	0	No	119	*Megasphaera* sp.	62
	7b	9	5.0	3	0	No	165	*L. iners*	54
	7c	8	5.5	2	1	No	130	*L. iners*	35
	7d	8	5.5	1	0	No	92	*Prevotella buccalis*	40

*Missing data. ^*#*^Long menstrual cycle.

**Table 2:** Relationship between BV score and the diversity, richness, and stability of the vaginal microbial community.

Participant^1^	BV score^2^	Simpson diversity (1D)	Chao1 species richness	Library similarity (%)^3^
1 (2)	0.5	0.15	7	97.0 ± 1.9
2 (2)	0	0.03	4	100.0 ± 0.1
3 (2)	3	0.38	14	79.1 ± 8.6
4 (3)	1	0.03	7	100 ± 0.1
5 (3)	1.3	0.33	15	91.0 ± 5.0
6 (4)	7	0.75	27	43.1 ± 4.6
7 (4)	8.3	0.85	22	38.2 ± 6.9

^1^Numbers in parentheses represent the total number of clone libraries for that participant. ^2^Mean of BV scores given in Table 1. ^3^Calculated by the nonparametric maximum likelihood estimator of Yue and Clayton. Values ± SE.

**Table 3:** Phylogenetic affiliation of 16S rRNA gene clones obtained from participants with and without bacterial vaginosis.

Phylum	Genus^1^	Participants with BV	Participants without BV	Total
*Firmicutes*				
	*Lactobacillus*	438	758	1196
	*Megasphaera*	180	0	180
	*Dialister*	41	0	41
	*Streptococcus*	3	19	22
	*Acetivibrio*	15	0	15
	*Aerococcus*	16	0	16
	*Micromonas*	8	0	8
	*Gemella*	5	1	6
	*Veillonella*	0	2	2
	*Anaerococcus*	1	1	2
	*Peptoniphilus*	0	1	1
	*Helcococcus*	1	0	1
	*Staphylococcus*	0	1	1
	*Turicibacter*	0	1	1

*Bacteroidetes*				
	*Prevotella^2^*	198	0	198

*Actinobacteria*				
	*Atopobium*	86	0	86
	*Cryptobacterium*	27	0	27
	*Eggerthella*	21	0	21
	*Gardnerella*	7	0	7
	*Mobiluncus*	5	0	5

*Proteobacteria*				
	*Escherichia*	0	1	30
	*Serratia*	2	0	23
	*Pseudomonas*	6	9	15
	*Janthinobacterium*	1	2	3
	*Ralstonia*	0	1	1
	*Dechloromonas*	0	1	1
	*Klebsiella*	0	1	1

*Fusobacteria*				
	*Sneathia*	83	0	83

Total		1144	799	1943
Total OTU_0.03_		31	19	46

^1^Typically, clones with >94% 16S rRNA gene identity to the nearest cultivated relative were considered members of that genus. ^2^Clones grouped with the *Prevotella* genus had 92–94% 16S rRNA gene identity to their closest cultivated relative. ^3^Operational taxonomic units (OTUs) were calculated at a cutoff of 97% similarity.
